# Efficacy and safety of quadruple therapy versus triple therapy in patients with heart failure with preserved ejection fraction: a propensity score-matched real-world study

**DOI:** 10.3389/fcvm.2026.1893975

**Published:** 2026-07-20

**Authors:** Jian Shi, Linglin Fang, Xiaoyue Cai, Hao Wu, Xiaoyu Yang, Liping Wei

**Affiliations:** 1 Graduate School, Tianjin Medical University, Tianjin, China; 2 School of Integrated Traditional Chinese and Western Medicine, Tianjin University of Traditional Chinese Medicine, Tianjin, China; 3 Department of Cardiology, Tianjin Union Medical Center, The First Affiliated Hospital of Nankai University, Nankai University, Tianjin, China

**Keywords:** beta-blockers, heart failure with preserved ejection fraction, propensity score matching, real-world study, triple therapy

## Abstract

**Objective:**

Although triple therapy has been recommended as foundational therapy for heart failure with preserved ejection fraction (HFpEF), residual risk remains high in affected patients. The efficacy of beta-blockers in HFpEF remains controversial.

**Methodology:**

This single-center retrospective cohort study included 600 patients with HFpEF who received triple therapy (as defined per the study protocol) at Tianjin Union Medical Center, The First Affiliated Hospital of Nankai University, between January 2018 and January 2025. A 1:1 propensity score matching (PSM) approach was used to balance baseline confounding factors. The efficacy and safety of quadruple therapy were compared with those of triple therapy alone. The primary endpoint was the composite of heart failure hospitalization or cardiovascular death.

**Results:**

After PSM, 360 patients were matched, with 180 patients in each group. During a median follow-up of 37.8 months, the incidence of the primary endpoint was significantly lower in the quadruple therapy group than in the triple therapy group [17.8% vs. 25.0%, hazard ratio (HR) = 0.678, 95% confidence interval (CI): 0.432–0.967, *P* = 0.035]. The quadruple therapy group had significantly lower rates of first heart failure hospitalization (14.4% vs. 20.6%, HR = 0.672, *P* = 0.030) and recurrent heart failure hospitalization (0.217 vs. 0.325 events per person-year, HR = 0.654, *P* = 0.015). Subgroup analyses showed that patients with concomitant coronary artery disease (HR = 0.591, *P* = 0.047) and baseline heart rate ≥70 beats per min (HR = 0.572, *P* = 0.045) derived greater benefit from quadruple therapy. A trend toward benefit was observed in patients with baseline LVEF of 50%–59%, but no significant interaction was detected. The overall safety profiles were comparable between the two groups.

**Conclusion:**

In this real-world observational cohort of patients with HFpEF receiving triple therapy, quadruple therapy was associated with a lower risk of the composite of heart failure hospitalization or cardiovascular death, driven primarily by a reduction in heart failure hospitalizations. No statistically significant difference in cardiovascular or all-cause mortality was observed. The association appeared more prominent in patients with concomitant coronary artery disease and higher baseline heart rate in exploratory subgroup analyses. Given the observational design, these hypothesis-generating findings cannot establish causality and require prospective validation.

## 1 Introduction

Heart failure with preserved ejection fraction (HFpEF) represents one of the major phenotypes of heart failure, accounting for over 50% of all heart failure cases globally (1). With population aging and the increasing prevalence of cardiovascular risk factors such as hypertension, diabetes mellitus, and obesity, the prevalence of HFpEF is rising annually, and the global number of patients with HFpEF is projected to increase by 25% by 2030 (2). A Chinese epidemiological survey of heart failure showed that the prevalence of HFpEF among residents aged ≥35 years in China is 1.1%, corresponding to an estimated 10 million affected individuals, making it a major public health concern (3). Compared with heart failure with reduced ejection fraction (HFrEF), HFpEF is associated with similarly poor prognosis, with a 5-year all-cause mortality rate of approximately 50% and higher rates of heart failure rehospitalization, imposing a substantial burden on healthcare systems (4).

Over the past two decades, breakthroughs have been made in the treatment of HFrEF, with the widespread use of neurohormonal inhibitors significantly improving patient outcomes. In contrast, the treatment of HFpEF has long lacked effective therapies, and traditional diuretics only relieve symptoms without reducing mortality (5). It was not until recent years that accumulating evidence from randomized controlled trials (RCTs) of sodium-glucose cotransporter 2 inhibitors (SGLT-2i), angiotensin receptor-neprilysin inhibitors (ARNI), and mineralocorticoid receptor antagonists (MRA) gradually established the foundational treatment regimen for HFpEF (6). Both the 2022 American College of Cardiology/American Heart Association/Heart Failure Society of America (ACC/AHA/HFSA) Guideline for the Management of Heart Failure and the 2023 Chinese Guidelines for the Diagnosis and Treatment of Heart Failure recommend the combination of SGLT-2i, MRA, and one agent from the ARNI, ACEI, or ARB class (used mutually exclusively), referred to as “triple therapy” as defined per the study protocol, for all patients with HFpEF (7, 8). RCTs such as EMPEROR-Preserved, PARAGON-HF, and FIDELIO-DKD have demonstrated that these three classes of drugs each reduce the risk of heart failure hospitalization or cardiovascular death by approximately 20%–30% in patients with HFpEF (9–11).

Despite the establishment of triple therapy as guideline-recommended foundational treatment, residual risk remains high in patients with HFpEF, with annual event rates reaching 8%–12% even with optimal foundational treatment (12). Therefore, exploring strategies to further optimize treatment regimens carries clinical relevance for patient management. Beta-blockers are cornerstone medications in the treatment of HFrEF, significantly reducing mortality and rehospitalization rates in affected patients (13). However, the efficacy of beta-blockers in HFpEF has long been controversial. The SENIORS trial, the first RCT to evaluate the efficacy of beta-blockers in elderly patients with heart failure, showed that nebivolol reduced all-cause mortality and cardiovascular hospitalization, but subgroup analyses suggested that the benefit was primarily derived from patients with LVEF <50% (14). Similar conclusions were reached in the CHARM-Preserved trial and the OPTIMIZE-HF registry study, indicating that beta-blockers may benefit patients with HFpEF and lower LVEF, but their efficacy in patients with LVEF ≥50% remains unclear (15, 16). A meta-analysis including 11 RCTs showed that beta-blockers reduced all-cause mortality but not heart failure hospitalization rates in patients with HFpEF (17).

Due to the strict inclusion and exclusion criteria of RCTs, the generalizability of their results is limited, and the real-world utilization and efficacy of beta-blockers in patients with HFpEF remain unclear. Multiple surveys have shown that the utilization rate of beta-blockers among patients with HFpEF in China is less than 50%, with even lower rates of dose titration to target (18). Furthermore, most previous studies did not use methods such as propensity score matching (PSM) to control for confounding factors, making it difficult to accurately assess the true efficacy of beta-blockers (19). Additionally, there is a lack of clear evidence regarding which subgroups of patients with HFpEF derive greater benefit from quadruple therapy.

This study employed a retrospective cohort study design and used a 1:1 nearest-neighbor matching PSM approach to balance baseline confounding factors. We compared the efficacy and safety of quadruple therapy versus triple therapy alone in patients with HFpEF, and explored the optimal population for beta-blocker therapy through subgroup analyses, aiming to provide real-world evidence for optimizing the treatment of HFpEF.

## 2 Materials and methods

### 2.1 Study design and participants

This was a single-center retrospective cohort study that consecutively enrolled patients with HFpEF who were diagnosed and treated in the inpatient and outpatient departments of the Department of Cardiology, Tianjin Union Medical Center, The First Affiliated Hospital of Nankai University, between January 1, 2018, and January 1, 2025. The index date was defined as the date when patients met all inclusion criteria and completed the baseline echocardiographic and laboratory assessment. Group assignment was determined solely by treatment status on the index date, adopting a prevalent user design. All included patients had received stable triple therapy (as defined per the study protocol) for at least 3 months before the index date. Patients in the quadruple therapy group initiated beta-blockers at least 3 months prior to the index date and maintained continuous use at baseline; patients who initiated beta-blockers during follow-up were excluded from the cohort to eliminate immortal time bias.

#### 2.1.1 Inclusion criteria

1) Met the diagnostic criteria for baseline HFpEF according to the 2023 Chinese Guidelines for the Diagnosis and Treatment of Heart Failure, applied uniformly via retrospective adjudication for all enrolled patients regardless of enrollment date: baseline left ventricular ejection fraction (LVEF) ≥50%; presence of heart failure-related symptoms and/or signs; N-terminal pro-B-type natriuretic peptide (NT-proBNP) ≥125 pg/mL; and echocardiographic evidence of left ventricular diastolic dysfunction (*E*/*e*′ ≥ 13 or left atrial diameter ≥40 mm). Patients who would not have met the 2023 diagnostic criteria were excluded, irrespective of whether they met earlier guideline definitions at the time of initial diagnosis. Dynamic changes in LVEF during follow-up were allowed, and patients were not excluded for this reason. 2) Had received triple therapy (as defined per the study protocol) for at least 3 months, including sodium-glucose cotransporter 2 inhibitors (SGLT-2i), angiotensin receptor-neprilysin inhibitors (ARNI) or angiotensin-converting enzyme inhibitors/angiotensin II receptor blockers (ACEI/ARB), and mineralocorticoid receptor antagonists (MRA). 3) Had complete clinical data and a follow-up duration of ≥12 months.

#### 2.1.2 Exclusion criteria

1) Baseline LVEF <50% (patients with LVEF decline during follow-up were retained). 2) Patients admitted for acute heart failure. 3) Presence of contraindications to beta-blocker use, including second-degree or higher atrioventricular block (without pacemaker implantation), acute exacerbation of bronchial asthma, sick sinus syndrome, and cardiogenic shock. 4) Concomitant malignant tumor with expected survival <1 year. 5) Concomitant severe liver disease (Child-Pugh class C). 6) Incomplete clinical data or loss to follow-up.

#### 2.1.3 Study population screening process

A total of 726 consecutive patients with HFpEF were screened, of whom 126 were excluded for not meeting the inclusion criteria or meeting the exclusion criteria:

Did not meet HFpEF diagnostic criteria: 32 cases; Admitted for acute heart failure: 28 cases; Presence of beta-blocker contraindications: 17 cases; Concomitant malignant tumor: 15 cases; Concomitant severe liver disease: 8 cases; Incomplete clinical data: 16 cases; Follow-up duration <12 months: 10 cases. Ultimately, 600 eligible patients with HFpEF were included in the study, with 220 patients in the quadruple therapy group and 380 patients in the triple therapy group.

#### 2.1.4 Baseline data collection

Baseline clinical data were collected from the hospital's electronic medical record system, including:

Demographic data: age, sex, body mass index (BMI), smoking history; Disease-related data: NYHA functional class, duration of heart failure, comorbidities (hypertension, type 2 diabetes mellitus, coronary artery disease, atrial fibrillation, chronic kidney disease); Physical examination data: systolic blood pressure, diastolic blood pressure, heart rate; Laboratory data: NT-proBNP, estimated glomerular filtration rate (eGFR), hemoglobin, serum potassium, fasting blood glucose, low-density lipoprotein cholesterol (LDL-C); Echocardiographic data: LVEF, left atrial diameter, *E*/*e*′ ratio, left ventricular end-diastolic diameter, peak tricuspid regurgitation velocity; Medication data: use of SGLT-2i, ARNI/ACEI/ARB, MRA, beta-blockers, statins, antiplatelet agents, and oral anticoagulants.

#### 2.1.5 LVEF measurement and follow-up assessment

Baseline LVEF was measured by echocardiography within 72 h of admission using the modified biplane Simpson's method. Echocardiography was repeated every 6 months during follow-up to record changes in LVEF. Prespecified subgroup analyses of LVEF were based exclusively on baseline LVEF measurements. The lowest LVEF value recorded during follow-up was used only for an exploratory *post-hoc* analysis. All echocardiographic examinations were independently performed by two experienced echocardiographers, with an intraclass correlation coefficient (ICC) of 0.92 (95% CI: 0.88–0.95) for LVEF measurement, indicating good interobserver agreement.

### 2.2 Grouping and treatment regimens

Patients were divided into two groups based exclusively on baseline treatment status on the index date, according to whether beta-blockers were used in addition to triple therapy (as defined per the study protocol): 1) Quadruple therapy group: Received SGLT-2i + a single RAAS inhibitor (either ARNI, ACEI, or ARB, used mutually exclusively) + MRA + beta-blockers; 2) Triple therapy group: Received only SGLT-2i + a single RAAS inhibitor (either ARNI, ACEI, or ARB, used mutually exclusively) + MRA, without any beta-blockers.

Beta-blocker use followed guideline recommendations, including metoprolol succinate extended-release tablets, bisoprolol fumarate, or carvedilol, initiated at low doses and gradually titrated to target doses or maximum tolerated doses. The target doses were: metoprolol succinate extended-release 190 mg once daily, bisoprolol fumarate 10 mg once daily, and carvedilol 25 mg twice daily. Dose titration was performed at the discretion of treating clinicians according to individual patient tolerance and clinical status, consistent with real-world clinical practice. In the quadruple therapy group, metoprolol succinate was prescribed in 112 patients (62.2%), bisoprolol fumarate in 54 patients (30.0%), and carvedilol in 14 patients (7.8%). The target-dose achievement rate was 62.8% at baseline, with a mean achieved daily dose of 71.4 ± 22.6% of the guideline-recommended target dose. During follow-up, dose down-titration due to adverse events occurred in 21 patients (11.7%), and permanent beta-blocker discontinuation occurred in 9 patients (5.0%). The 12-month treatment persistence rate was 94.4%. Detailed drug-specific dosing, reasons for dose adjustment and discontinuation, and long-term treatment persistence data are summarized in Supplementary Table S4. All beta-blockers were initiated at least 3 months before the index date, and the baseline dose represented the stable maintenance dose at study entry. Beta-blocker persistence was defined as continuous use without interruption exceeding 30 days during follow-up. Discontinuation was defined as permanent cessation of beta-blocker therapy for at least 30 days, irrespective of cause. Dose adjustment included up-titration toward target dose, down-titration, or temporary suspension due to adverse events or clinical deterioration. Medication adherence, dose changes, and discontinuation events were documented at each scheduled follow-up via electronic medical record review and patient interview. Treatment for other underlying diseases in all patients was administered according to relevant guidelines. All patients received loop diuretics as needed for symptom control according to clinical practice.

### 2.3 Propensity score matching (PSM)

To control for the influence of baseline confounding factors on study results, a 1:1 nearest-neighbor matching method was used for propensity score matching with a caliper width of 0.05. Matching variables included all baseline clinical characteristics listed in Table 1: age, sex, BMI, smoking history, NYHA functional class, duration of heart failure, systolic blood pressure, diastolic blood pressure, baseline heart rate, history of hypertension, history of type 2 diabetes mellitus, history of coronary artery disease, history of atrial fibrillation, history of chronic kidney disease, baseline NT-proBNP level, eGFR, hemoglobin, serum potassium, fasting blood glucose, LDL-C, baseline LVEF, left atrial diameter, *E*/*e*′ ratio, left ventricular end-diastolic diameter, peak tricuspid regurgitation velocity, history of statin use, history of antiplatelet agent use, and history of oral anticoagulant use. Matching was based solely on baseline characteristics and did not include changes in LVEF during follow-up. Standardized mean differences (SMD) were calculated for all covariates before and after matching to evaluate covariate balance, with SMD <0.1 defined as negligible imbalance. The distribution of propensity scores before and after matching was visualized via histograms, and a Love plot was generated to graphically display SMD values across all covariates. Full balance diagnostics are presented in Supplementary Table S2 and Figures S1, S2.

**Table 1:** Baseline clinical characteristics of patients with hFpEF before and after propensity score matching.

Characteristic	Before PSM			After PSM		
	Quadruple Therapy (*n* = 220)	Triple Therapy (*n* = 380)	*P*-value	Quadruple Therapy (*n* = 180)	Triple Therapy (*n* = 180)	*P*-value
Age (years), *x¯* ± *s*	70.8 ± 8.7	69.7 ± 9.2	0.028	70.5 ± 8.5	70.2 ± 8.9	0.742
Female, *n* (%)	120 (54.5)	217 (57.1)	0.543	95 (52.8)	96 (53.3)	0.916
BMI (kg/m^2^), *x¯* ± *s*	28.6 ± 4.2	27.8 ± 4.6	0.295	28.0 ± 3.9	27.9 ± 4.3	0.819
Obesity (BMI ≥30 kg/m^2^), *n* (%)	94 (42.7)	146 (38.4)	0.299	72 (40.0)	70 (38.9)	0.829
Smoking history, *n* (%)						
Current smoker	32 (14.5)	62 (16.3)		26 (14.4)	25 (13.9)	
Former smoker	76 (34.5)	121 (31.8)		62 (34.4)	61 (33.9)	
Never smoker	112 (50.9)	197 (51.8)	0.736	92 (51.1)	94 (52.2)	0.976
NYHA functional class, *n* (%)						
Class II	82 (37.3)	162 (42.6)		71 (39.4)	74 (41.1)	
Class III	119 (54.1)	187 (49.2)		92 (51.1)	89 (49.4)	
Class IV	19 (8.6)	31 (8.2)	0.433	17 (9.4)	17 (9.4)	0.946
HF duration (years), *x¯* ± *s*	3.1 ± 2.2	2.9 ± 1.7	0.276	3.0 ± 2.1	3.0 ± 1.9	0.963
Systolic blood pressure (mmHg), *x¯* ± *s*	139.5 ± 15.3	136.2 ± 14.4	0.036	138.7 ± 14.9	137.8 ± 14.2	0.847
Diastolic blood pressure (mmHg), *x¯* ± *s*	79.2 ± 9.6	76.6 ± 8.8	0.007	78.0 ± 9.3	77.8 ± 9.4	0.836
Heart rate (beats/min), *x¯* ± *s*	76.2 ± 10.5	75.8 ± 10.2	0.654	76.0 ± 10.3	75.9 ± 10.4	0.925
Hypertension, *n* (%)	200 (90.9)	339 (89.2)	0.507	163 (90.6)	161 (89.4)	0.725
Type 2 diabetes mellitus, *n* (%)	104 (47.3)	161 (42.4)	0.244	81 (45.0)	79 (43.9)	0.832
Coronary artery disease, *n* (%)	127 (57.7)	171 (45.0)	0.003	93 (51.7)	90 (50.0)	0.752
Atrial fibrillation, *n* (%)	87 (39.5)	139 (36.6)	0.470	70 (38.9)	69 (38.3)	0.914
Chronic kidney disease (eGFR < 60 mL/min/1.73m^2^), *n* (%)	80 (36.4)	123 (32.4)	0.319	64 (35.6)	62 (34.4)	0.825
NT-proBNP (pg/mL), M(P25,P75)	1792 (963, 3187)	1728 (912, 3056)	0.431	1765 (948, 3124)	1736 (925, 3078)	0.792
eGFR (mL/min/1.73m^2^), *x¯* ± *s*	64.5 ± 18.4	66.0 ± 19.7	0.387	65.1 ± 18.2	65.5 ± 18.9	0.833
Hemoglobin (g/L), *x¯* ± *s*	128.2 ± 15.5	130.0 ± 14.9	0.179	128.9 ± 14.8	129.5 ± 14.6	0.698
Serum potassium (mmol/L), *x¯* ± *s*	4.20 ± 0.41	4.17 ± 0.45	0.428	4.19 ± 0.42	4.18 ± 0.43	0.818
Fasting blood glucose (mmol/L), *x¯* ± *s*	6.84 ± 2.17	6.73 ± 2.01	0.702	6.77 ± 2.14	6.75 ± 2.12	0.926
LDL-C (mmol/L), *x¯* ± *s*	2.62 ± 0.89	2.67 ± 0.93	0.518	2.64 ± 0.87	2.66 ± 0.90	0.824
LVEF (%), *x¯* ± *s*	54.7 ± 3.3	56.8 ± 4.6	0.021	55.9 ± 4.2	56.0 ± 4.4	0.817
Left atrial diameter (mm), *x¯* ± *s*	42.2 ± 4.6	41.7 ± 5.4	0.279	42.0 ± 5.1	41.9 ± 5.3	0.848
*E*/*e*′ ratio, *x¯* ± *s*	15.1 ± 3.9	14.8 ± 3.5	0.358	15.0 ± 3.8	14.9 ± 3.7	0.801
Left ventricular end-diastolic diameter (mm), *x¯* ± *s*	48.2 ± 5.3	47.9 ± 5.7	0.536	48.1 ± 5.2	48.0 ± 5.4	0.856
Peak tricuspid regurgitation velocity (m/s), *x¯* ± *s*	2.71 ± 0.46	2.67 ± 0.44	0.298	2.69 ± 0.45	2.68 ± 0.44	0.822
SGLT-2 inhibitors, *n* (%)	220 (100.0)	380 (100.0)	1.000	180 (100.0)	180 (100.0)	1.000
ARNI/ACEI/ARB and MRA, *n* (%)	220 (100.0)	380 (100.0)	1.000	180 (100.0)	180 (100.0)	1.000
Loop diuretics, *n* (%)	220 (100.0)	380 (100.0)	1.000	180 (100.0)	180 (100.0)	1.000
Beta-blockers, *n* (%)	220 (100.0)	0 (0.0)	0.000	180 (100.0)	0 (0.0)	0.000
Statins, *n* (%)	164 (74.5)	272 (71.6)	0.432	133 (73.9)	131 (72.8)	0.812
Antiplatelet agents, *n* (%)	118 (53.6)	156 (41.1)	0.003	91 (50.6)	89 (49.4)	0.833
Oral anticoagulants, *n* (%)	71 (32.3)	108 (28.4)	0.320	57 (31.7)	55 (30.6)	0.820

BMI, body mass index; HF, heart failure; NYHA, New York Heart Association; eGFR, estimated glomerular filtration rate; NT-proBNP, N-terminal pro-B-type natriuretic peptide; LDL-C, low-density lipoprotein cholesterol; LVEF, left ventricular ejection fraction; SGLT-2i, sodium-glucose cotransporter 2 inhibitor; ARNI, angiotensin receptor-neprilysin inhibitor; ACEI, angiotensin-converting enzyme inhibitor; ARB, angiotensin II receptor blocker; these three drug classes were used mutually exclusively. MRA, mineralocorticoid receptor antagonist.

### 2.4 Outcome measures

Primary endpoint: The primary composite endpoint was heart failure hospitalization or cardiovascular death. Heart failure hospitalization was defined as hospitalization for worsening heart failure symptoms with the primary treatment being intensive heart failure therapy during hospitalization. Cardiovascular death was defined as death due to myocardial infarction, heart failure, arrhythmia, stroke, or other cardiovascular causes.

Secondary endpoints: Cardiovascular death, all-cause death, first heart failure hospitalization, total number of heart failure rehospitalizations and rehospitalization rate (events per person-year), percentage reduction in NT-proBNP from baseline at 12 and 24 months of follow-up, and change in eGFR from baseline at 24 months of follow-up.

Safety endpoints: All adverse events occurring during follow-up were recorded, including: 1) Cardiovascular adverse events: symptomatic hypotension, sinus bradycardia (heart rate <50 beats per min), atrioventricular block, exacerbation of atrial fibrillation. 2) Electrolyte and renal adverse events: hyperkalemia (serum potassium >5.5 mmol/L), acute kidney injury [defined according to Kidney Disease: Improving Global Outcomes (KDIGO) criteria].

Other systemic adverse events: fatigue/asthenia, dizziness/headache, gastrointestinal reactions, bronchospasm, hypoglycemia. 3) The severity of adverse events was graded according to the National Cancer Institute Common Terminology Criteria for Adverse Events (NCI-CTCAE) version 5.0. Adverse events were systematically ascertained at each prespecified follow-up visit via standardized symptom inquiry and electronic medical record review, with identical ascertainment protocols applied to both treatment groups. All safety outcomes were independently adjudicated by two cardiologists blinded to treatment assignment, following the same adjudication workflow as efficacy endpoints. Disagreements were resolved through consensus review by a third senior cardiologist.

### 2.5 Follow-up

All patients were followed up through outpatient visits, telephone interviews, and inpatient medical record review, with a cutoff date of May 1, 2026. Follow-up frequency was every 3 months for the first 2 years after treatment initiation, and every 6 months thereafter. Follow-up content included heart failure symptoms and signs, medication adherence, occurrence of adverse events, and laboratory test results (NT-proBNP, renal function, electrolytes, etc.). Endpoint events were independently adjudicated by two cardiologists blinded to treatment group assignment. Disagreements were resolved by a third senior cardiologist through consensus review. The median follow-up duration was 37.8 months (IQR: 26.4–51.2 months), with a minimum follow-up of 12.1 months and a maximum follow-up of 72.3 months.

### 2.6 Statistical analysis

Statistical analyses were performed using SPSS (Statistical Package for the Social Sciences) version 26.0 and R 4.3.1 software. Normally distributed continuous variables were expressed as mean ± standard deviation (*x¯* ± *s*), and between-group comparisons were performed using the independent samples t-test. Skewed continuous variables were expressed as median (interquartile range) [M(P25,P75)], and between-group comparisons were performed using the Mann–Whitney *U* test. Categorical variables were expressed as number (percentage) [*n*(%)], and between-group comparisons were performed using the *χ*^2^ test or Fisher's exact test. Kaplan–Meier method was used to plot survival curves, and between-group comparisons of survival rates were performed using the Log-rank test. Cox proportional hazards regression model was used to analyze independent risk factors for the primary endpoint. The proportional hazards assumption for all Cox models was verified using Schoenfeld residual tests. For recurrent heart failure hospitalizations, the Andersen-Gill model, a standard method for time-to-event analysis with multiple recurring events, was applied to estimate hazard ratios by incorporating all hospitalization episodes and accounting for the time order of events, with event rates per person-year presented as descriptive supplements. Given the limited number of endpoint events, two multivariable Cox models were constructed to mitigate overfitting risk. First, a backward stepwise Cox regression was conducted, with variables showing *P* < 0.1 in univariate analysis entered into the selection procedure and a retention significance level of 0.05. This data-driven approach was adopted for exploratory identification of independent prognostic factors in the study cohort. To address the potential instability of stepwise variable selection, a second prespecified multivariable model was built including seven clinically established prognostic factors for HFpEF (age, NYHA functional class, coronary artery disease, chronic kidney disease, baseline NT-proBNP, eGFR, and *E*/*e*′ ratio), to verify the stability of the treatment effect independent of data-driven variable selection. Prespecified subgroup analyses were all based on baseline characteristics, including sex, baseline LVEF, coronary artery disease status, baseline heart rate, atrial fibrillation status, baseline eGFR, and type 2 diabetes mellitus status, with interaction tests performed to assess heterogeneity of treatment effects across subgroups. All subgroup analyses were exploratory in nature and not adjusted for multiple testing; results should be interpreted as hypothesis-generating rather than confirmatory, and cannot independently support clinical recommendations. In addition, an exploratory *post-hoc* subgroup analysis was conducted using the lowest LVEF recorded during follow-up to explore the potential association between LVEF trajectory and treatment effect. This *post-hoc* analysis was hypothesis-generating and not prespecified. To evaluate the robustness of primary findings and address potential residual confounding and confounding by indication, two prespecified sensitivity analyses were conducted. The primary analysis followed an intention-to-treat-like principle based on baseline treatment assignment; as-treated analysis was not adopted as the primary analytical approach to avoid time-dependent bias introduced by post-baseline treatment changes. First, inverse probability of treatment weighting (IPTW) was applied using the identical set of covariates included in the PSM model, with stabilized weights to reduce estimation variance. Weighted standardized mean differences were calculated to verify covariate balance after weighting (detailed in Supplementary Table S1). Second, a 12-month landmark analysis was performed, restricted to patients who remained free of primary endpoint events and maintained their original treatment assignment at 12 months after the index date, to further mitigate bias related to early events and treatment changes during follow-up. All statistical tests were two-sided, and a *P*-value <0.05 was considered statistically significant.

## 3 Results

### 3.1 Baseline characteristics of the study population

A total of 726 consecutive patients with HFpEF were screened, and 126 were excluded for not meeting the eligibility criteria, resulting in a final cohort of 600 patients, including 220 in the quadruple therapy group and 380 in the triple therapy group. Before PSM, significant differences were observed between the two groups in age (*P* = 0.028), systolic blood pressure (*P* = 0.036), diastolic blood pressure (*P* = 0.007), prevalence of coronary artery disease (*P* = 0.003), LVEF (*P* = 0.021), and antiplatelet agent use (*P* = 0.003). After 1:1 nearest-neighbor matching with a caliper width of 0.05, 360 patients were successfully matched, with 180 patients in each group. After matching, the standardized mean differences (SMD) for all baseline characteristics were <0.1, indicating well-balanced baseline characteristics between the two groups (Table 1). Full SMD values for all covariates, propensity score distribution histograms, and the Love plot for PSM balance diagnostics are presented in Supplementary Table S2 and Figures S1, S2, respectively.

### 3.2 Primary endpoint events

**Figure 1:**
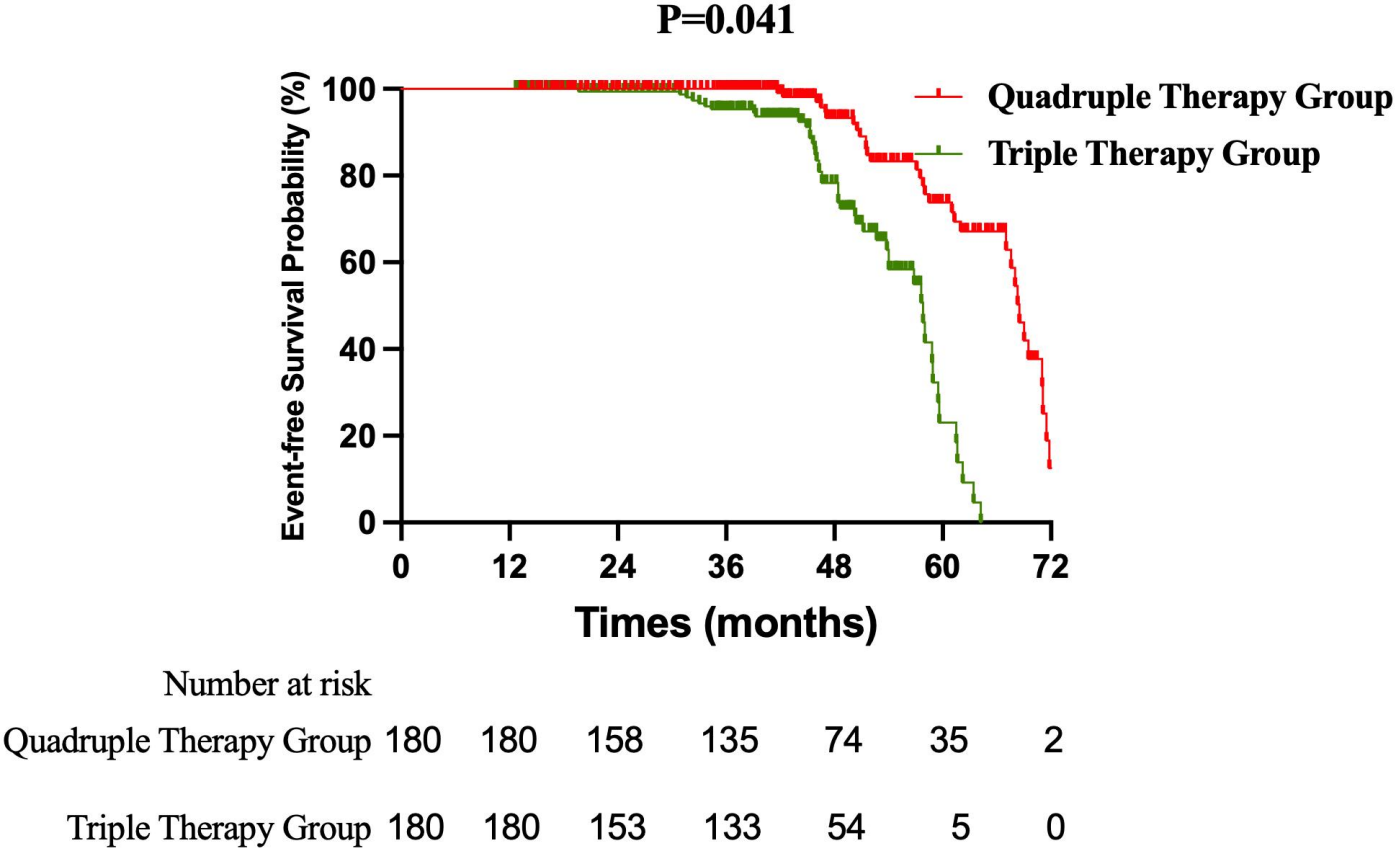
Kaplan–Meier survival curves for the primary composite endpoint. Median follow-up: 37.8 months (range: 12.1–72.3 months). Quadruple Therapy Group vs. Triple Therapy Group. Log-rank *χ*^2^ = 4.03, *P* = 0.041. HR = 0.678, 95% CI: 0.432–0.967.

During a median follow-up of 37.8 months (IQR: 26.4–51.2 months), 32 primary endpoint events (17.8%) occurred in the quadruple therapy group, compared with 45 events (25.0%) in the triple therapy group. The absolute risk reduction for the primary composite endpoint was 7.2% in the quadruple therapy group, corresponding to a number needed to treat of 14 over the median follow-up period. Kaplan–Meier survival analysis showed that the event-free survival rate was significantly higher in the quadruple therapy group than in the triple therapy group (Log-rank *χ*^2^ = 4.03, *P* = 0.041) (Figure 1). Univariate Cox regression analysis showed that quadruple therapy was associated with a significantly lower risk of primary endpoint events compared with triple therapy (HR = 0.678, 95% CI: 0.432–0.967, *P* = 0.035) (Table 2).

**Table 2:** Comparison of efficacy endpoints between the Two groups after PSM.

Efficacy endpoint	Quadruple Therapy (*n* = 180)	Triple Therapy (*n* = 180)	HR (95% CI)	*P*-value
Primary endpoint				
Composite of HF hospitalization or cardiovascular death, *n* (%)	32 (17.8)	45 (25.0)	0.678 (0.432–0.967)	0.035
Secondary endpoints				
Cardiovascular death, *n* (%)	11 (6.1)	16 (8.9)	0.761 (0.418–1.475)	0.365
All-cause death, *n* (%)	15 (8.3)	20 (11.1)	0.719 (0.372–1.596)	0.312
First HF hospitalization, *n* (%)	26 (14.4)	37 (20.6)	0.672 (0.403–0.997)	0.030
Total number of HF rehospitalizations, events	42	68	—	—
Changes in cardiac function parameters				
NT-proBNP reduction at 12 months, %, *x¯* ± *s*	38.2 ± 15.4	29.5 ± 14.3	—	0.002
NT-proBNP reduction at 24 months, %, *x¯* ± *s*	42.6 ± 17.2	31.8 ± 15.9	—	<0.001
Change in eGFR at 24 months, mL/min/1.73m^2^, *x¯* ± *s*	-2.1 ± 3.5	-2.3 ± 3.8	—	0.587
Cumulative event rates				
6-month composite endpoint rate, %	3.9	5.0	—	0.628
12-month composite endpoint rate, %	8.9	12.5	—	0.215
18-month composite endpoint rate, %	13.3	19.4	—	0.107
24-month composite endpoint rate, %	17.8	25.0	—	0.043

HF, heart failure; HR, hazard ratio; CI, confidence interval; NT-proBNP, N-terminal pro-B-type natriuretic peptide; eGFR, estimated glomerular filtration rate.

Sensitivity Analyses: Consistent results were observed in both sensitivity analyses. In the IPTW analysis (weighted analytical cohort *n* = 592), standardized mean differences of all covariates remained below 0.1 after weighting, indicating adequate covariate balance (Supplementary Table S1). Quadruple therapy was associated with a lower risk of the primary composite endpoint, with a direction of effect consistent with the primary PSM analysis (HR = 0.701, 95% CI: 0.462–1.064, *P* = 0.093). In the 12-month landmark analysis restricted to event-free patients at 12 months (*n* = 317), the association remained consistent in direction (HR = 0.643, 95% CI: 0.391–1.057, *P* = 0.082). The consistent effect direction across analytical approaches supports the robustness of the primary findings. A calendar-period sensitivity analysis stratified by enrollment period (2018–2020 vs. 2021–2025) showed a consistent direction of treatment association across periods, with no significant interaction detected (*P* for interaction >0.05). This finding indicates that temporal changes in clinical practice did not materially affect the study results.

### 3.3 Secondary endpoint events

There were no statistically significant differences between the two groups in cardiovascular death (6.1% vs. 8.9%, HR = 0.761, 95% CI: 0.418–1.475, *P* = 0.365) and all-cause death (8.3% vs. 11.1%, HR = 0.719, 95% CI: 0.372–1.596, *P* = 0.312). The rate of first heart failure hospitalization was significantly lower in the quadruple therapy group than in the triple therapy group (14.4% vs. 20.6%, HR = 0.672, 95% CI: 0.403–0.997, *P* = 0.030). During follow-up, there were 42 heart failure rehospitalizations in the quadruple therapy group, corresponding to a rehospitalization rate of 0.217 events per person-year, compared with 68 rehospitalizations and a rate of 0.325 events per person-year in the triple therapy group. The heart failure rehospitalization rate was significantly lower in the quadruple therapy group, as estimated by the Andersen-Gill recurrent events model (HR = 0.654, 95% CI: 0.384–0.919, *P* = 0.015) (Table 2).

### 3.4 Changes in cardiac function parameters

**Figure 2:**
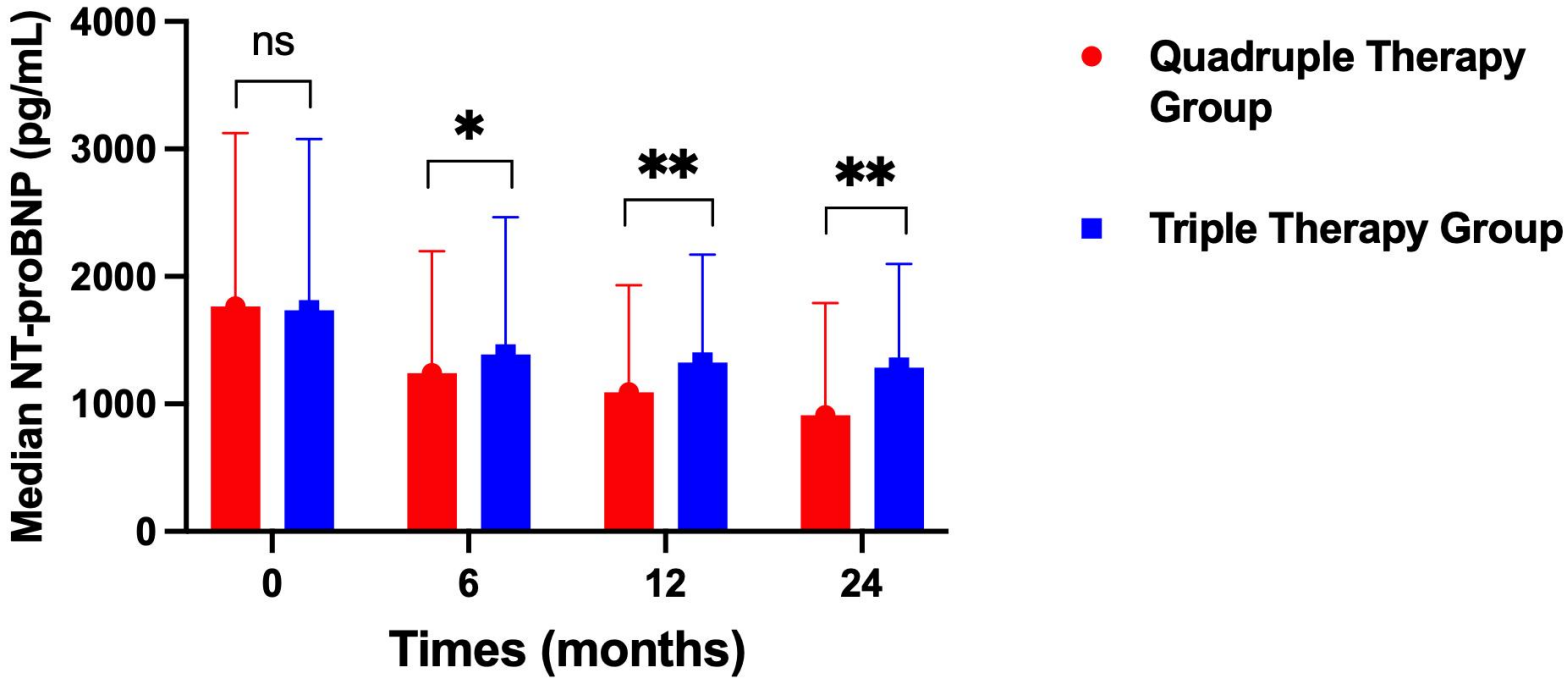
Changes in median NT-proBNP levels during follow-up.

There was no statistically significant difference in baseline NT-proBNP levels between the two groups (1,765 pg/mL vs. 1,736 pg/mL, *P* = 0.792). At 6 months of follow-up, the median NT-proBNP level was already lower in the quadruple therapy group than in the triple therapy group (1,243 pg/mL vs. 1,389 pg/mL, *P* = 0.042); the difference between the two groups further widened at 12 and 24 months of follow-up (1,091 pg/mL vs. 1,324 pg/mL, *P* = 0.007; 913 pg/mL vs. 1,284 pg/mL, *P* = 0.002) (Figure 2). Compared with baseline, the percentage reductions in NT-proBNP at 12 and 24 months of follow-up were significantly greater in the quadruple therapy group than in the triple therapy group (38.2 ± 15.4% vs. 29.5 ± 14.3%, *P* = 0.002; 42.6 ± 17.2% vs. 31.8 ± 15.9%, *P* < 0.001). There was no statistically significant difference in the change in eGFR from baseline at 24 months of follow-up between the two groups (−2.1 ± 3.5 mL/min/1.73m^2^ vs. −2.3 ± 3.8 mL/min/1.73m^2^, *P* = 0.587) (Table 2).

### 3.5 Safety endpoints

There were no statistically significant differences between the two groups in the overall incidence of adverse events (28.9% vs. 25.6%, *P* = 0.477), incidence of serious adverse events (6.1% vs. 5.0%, *P* = 0.645), and rate of treatment discontinuation due to adverse events (5.0% vs. 3.9%, *P* = 0.609). Among cardiovascular adverse events, the incidence of sinus bradycardia (heart rate <50 beats per min) was significantly higher in the quadruple therapy group than in the triple therapy group (6.7% vs. 2.2%, *P* = 0.041). All cases were grade 1–2 per NCI-CTCAE 5.0 criteria, and none were symptomatic or required pacemaker implantation. Events resolved after beta-blocker dose reduction or temporary interruption, with no cases of severe bradycardia or cardiac arrest reported. There were no statistically significant differences between the two groups in the incidence of symptomatic hypotension, atrioventricular block, exacerbation of atrial fibrillation, hyperkalemia, acute kidney injury, and other systemic adverse events (Table 3).

**Table 3:** Comparison of safety endpoints between the Two groups after PSM.

Adverse event	Quadruple therapy (*n* = 180)	Triple therapy (*n* = 180)	*P*-value
Overall adverse events			
Any adverse event, *n* (%)	52 (28.9)	46 (25.6)	0.477
Serious adverse events (≥Grade 3), *n* (%)	11 (6.1)	9 (5.0)	0.645
Drug-related adverse events, *n* (%)	38 (21.1)	31 (17.2)	0.349
Treatment discontinuation due to adverse events, *n* (%)	9 (5.0)	7 (3.9)	0.609
Cardiovascular adverse events			
Symptomatic hypotension, *n* (%)	15 (8.3)	10 (5.6)	0.300
Sinus bradycardia (<50 beats/min), *n* (%)	12 (6.7)	4 (2.2)	0.041
First-degree atrioventricular block, *n* (%)	7 (3.9)	2 (1.1)	0.174
Second-degree or higher atrioventricular block, *n* (%)	2 (1.1)	0 (0.0)	0.499
Exacerbation of atrial fibrillation, *n* (%)	4 (2.2)	5 (2.8)	1.000
Electrolyte and renal adverse events			
Hyperkalemia, *n* (%)	12 (6.7)	10 (5.6)	0.660
Mild hyperkalemia (5.5–6.0 mmol/L)	9 (5.0)	6 (3.3)	0.429
Moderate hyperkalemia (6.0–6.5 mmol/L)	3 (1.7)	2 (1.1)	1.000
Severe hyperkalemia (>6.5 mmol/L)	0 (0.0)	0 (0.0)	1.000
Acute kidney injury, *n* (%)	8 (4.4)	5 (2.8)	0.397
Acute kidney injury Stage 1	7 (3.9)	4 (2.2)	0.358
Acute kidney injury Stages 2–3	1 (0.6)	1 (0.6)	1.000
Other systemic adverse events			
Fatigue/asthenia, *n* (%)	9 (5.0)	6 (3.3)	0.429
Dizziness/headache, *n* (%)	7 (3.9)	5 (2.8)	0.557
Gastrointestinal reactions (nausea/diarrhea), *n* (%)	6 (3.3)	7 (3.9)	0.778
Bronchospasm, *n* (%)	2 (1.1)	1 (0.6)	0.561
Hypoglycemia, *n* (%)	3 (1.7)	1 (1.1)	0.623

### 3.6 Multivariate Cox regression analysis of factors influencing the primary endpoint

Variables with *P* < 0.1 in univariate analysis (treatment group, age, NYHA class III/IV, heart rate, coronary artery disease, atrial fibrillation, type 2 diabetes mellitus, chronic kidney disease, NT-proBNP, eGFR, LVEF, *E*/*e*′ ratio) were included in the multivariate stepwise Cox regression analysis. The results showed that quadruple therapy was an independent protective factor for primary endpoint events in patients with HFpEF (HR = 0.652, 95% CI: 0.413–0.943, *P* = 0.024). In addition, age (per 10-year increase, HR = 1.283, 95% CI: 1.012–1.625, *P* = 0.041), NYHA class III/IV (HR = 1.987, 95% CI: 1.218–3.247, *P* = 0.007), coronary artery disease (HR = 1.656, 95% CI: 1.035–2.640, *P* = 0.037), chronic kidney disease (HR = 1.716, 95% CI: 1.107–2.825, *P* = 0.019), baseline NT-proBNP (per 1,000 pg/mL increase, HR = 1.188, 95% CI: 1.059–1.336, *P* = 0.005), and *E*/*e*′ ratio (per 5-unit increase, HR = 1.272, 95% CI: 1.048–1.554, *P* = 0.018) were independent risk factors for primary endpoint events (Table 4). In the prespecified multivariable Cox model adjusted for seven established prognostic factors, quadruple therapy remained independently associated with a reduced risk of the primary endpoint (HR = 0.664, 95% CI: 0.419–0.987, *P* = 0.043), consistent with the result of the stepwise model. Schoenfeld residual tests confirmed that the proportional hazards assumption was satisfied for both models (all global *P* values >0.05).

**Table 4:** Univariate and multivariate Cox regression analysis of factors influencing the primary endpoint.

Variable	Univariate cox regression		Multivariate cox regression (stepwise)	
	HR (95% CI)	*P*-value	HR (95% CI)	*P*-value
Treatment group (Quadruple vs. Triple)	0.678 (0.432, 0.967)	0.035	0.652 (0.413, 0.943)	0.024
Age (per 10-year increase)	1.324 (1.052, 1.663)	0.017	1.283 (1.012, 1.625)	0.041
Female (vs. Male)	0.923 (0.583, 1.465)	0.721	—	—
BMI (per 5 kg/m^2^ increase)	1.154 (0.895, 1.484)	0.287	—	—
Current smoker (vs. Never smoker)	1.426 (0.763, 2.652)	0.268	—	—
Former smoker (vs. Never smoker)	1.183 (0.712, 1.964)	0.523	—	—
NYHA class III/IV (vs. Class II)	2.153 (1.324, 3.512)	0.002	1.987 (1.218, 3.247)	0.007
HF duration (per 1-year increase)	1.085 (0.973, 1.203)	0.156	—	—
Systolic blood pressure (per 10 mmHg increase)	0.953 (0.826, 1.102)	0.489	—	—
Heart rate (per 10 beats/min increase)	1.266 (1.032, 1.543)	0.025	1.227 (0.994, 1.509)	0.061
Coronary artery disease (vs. No)	1.788 (1.125, 2.835)	0.015	1.656 (1.035, 2.640)	0.037
Atrial fibrillation (vs. No)	1.450 (0.916, 2.316)	0.118	1.387 (0.862, 2.226)	0.179
Type 2 diabetes mellitus (vs. No)	1.527 (0.967, 2.418)	0.074	1.471 (0.923, 2.355)	0.106
Chronic kidney disease (vs. No)	1.895 (1.192, 3.006)	0.007	1.716 (1.107, 2.825)	0.019
NT-proBNP (per 1,000 pg/mL increase)	1.214 (1.080, 1.363)	0.001	1.188 (1.059, 1.336)	0.005
eGFR (per 10 mL/min/1.73m^2^ decrease)	1.176 (1.028, 1.453)	0.026	1.128 (0.984, 1.304)	0.089
LDL-C (per 1 mmol/L increase)	1.054 (0.819, 1.346)	0.698	—	—
Serum potassium (per 0.5 mmol/L increase)	1.120 (0.874, 1.445)	0.365	—	—
LVEF (per 5% decrease)	1.247 (1.031, 1.492)	0.022	1.203 (0.995, 1.458)	0.063
*E*/*e*′ ratio (per 5-unit increase)	1.323 (1.094, 1.603)	0.004	1.272 (1.048, 1.554)	0.018
Left atrial diameter (per 5 mm increase)	1.196 (0.967, 1.479)	0.112	—	—
Peak tricuspid regurgitation velocity (per 0.5 m/s increase)	1.214 (0.935, 1.577)	0.153	—	—

Variables with *P* < 0.1 in univariate analysis were included in the multivariate stepwise Cox regression analysis. A backward elimination method was used with a significance level of 0.05 for retention in the final model.

### 3.7 Subgroup analyses

**Figure 3:**
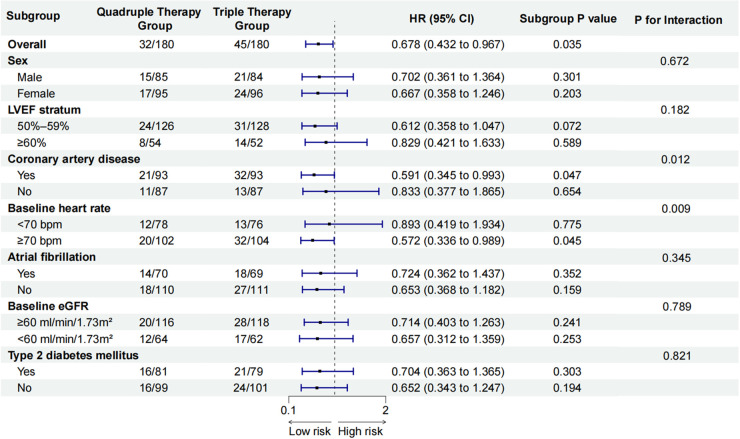
Forest plot of subgroup analyses for the primary endpoint.

Subgroup analyses showed no statistically significant differences in the efficacy of quadruple therapy across subgroups defined by sex, atrial fibrillation status, baseline eGFR level, and type 2 diabetes mellitus status (all *P* for interaction >0.05). In the prespecified subgroup analysis stratified by baseline LVEF (50%–59% vs. ≥60%), no significant heterogeneity of treatment effect was observed (*P* for interaction = 0.182). Patients with baseline LVEF of 50%–59% showed a trend toward lower primary endpoint risk with quadruple therapy (HR = 0.612, 95% CI: 0.358–1.047, *P* = 0.072), while no significant association was detected in those with baseline LVEF ≥60% (HR = 0.829, 95% CI: 0.421–1.633, *P* = 0.589). Significant heterogeneity was observed in subgroups stratified by coronary artery disease status and baseline heart rate (*P* for interaction = 0.012 and 0.009, respectively): patients with concomitant coronary artery disease (HR = 0.591, 95% CI: 0.345–0.993, *P* = 0.047) and baseline heart rate ≥70 beats per min (HR = 0.572, 95% CI: 0.336–0.989, *P* = 0.045) showed a more pronounced association between quadruple therapy and reduced primary endpoint risk (Table 5, Figure 3). Given the limited sample size of individual strata and the absence of adjustment for multiple comparisons, these interaction test results should be interpreted with caution and do not represent confirmatory evidence.

**Table 5:** Subgroup analysis of the effect of quadruple therapy on the primary endpoint.

Subgroup	Subgroup definition	Events/total (quadruple)	Events/total (triple)	HR (95% CI)	Subgroup *P*-value	*P* for interaction
Overall population	—	32/180	45/180	0.678 (0.432, 0.967)	0.035	—
Sex	Male	15/85	21/84	0.702 (0.361, 1.364)	0.301	0.672
	Female	17/95	24/96	0.667 (0.358, 1.246)	0.203	
Baseline LVEF stratum	50%–59%	24/126	31/128	0.612 (0.358–1.047)	0.072	0.182
	≥60%	8/54	14/52	0.829 (0.421–1.633)	0.589	
Coronary artery disease	Yes	21/93	32/93	0.591 (0.345, 0.993)	0.047	0.012
	No	11/87	13/87	0.833 (0.377, 1.865)	0.654	
Baseline heart rate	<70 bpm	12/78	13/76	0.893 (0.419, 1.934)	0.775	0.009
	≥70 bpm	20/102	32/104	0.572 (0.336, 0.989)	0.045	
Atrial fibrillation	Yes	14/70	18/69	0.724 (0.362, 1.437)	0.352	0.345
	No	18/110	27/111	0.653 (0.368, 1.182)	0.159	
Baseline eGFR	≥60 mL/min/1.73m^2^	20/116	28/118	0.714 (0.403, 1.263)	0.241	0.789
	<60 mL/min/1.73m^2^	12/64	17/62	0.657 (0.312, 1.359)	0.253	
Type 2 diabetes mellitus	Yes	16/81	21/79	0.704 (0.363, 1.365)	0.303	0.821
	No	16/99	24/101	0.652 (0.343, 1.247)	0.194	

All prespecified subgroup analyses were based on baseline characteristics. All patients had baseline LVEF ≥50%.

Exploratory *post-hoc* Analysis by Follow-up Lowest LVEF: An exploratory *post-hoc* analysis was performed by stratifying patients according to the lowest LVEF value recorded during follow-up. In this analysis, apparent heterogeneity of treatment effect was observed across LVEF strata (*P* for interaction = 0.028). Patients with follow-up lowest LVEF of 40%–49% showed the strongest association between quadruple therapy and reduced endpoint risk (HR = 0.421, 95% CI: 0.183–0.976, *P* = 0.044), while no significant association was detected in patients with follow-up lowest LVEF ≥60% (HR = 0.954, 95% CI: 0.285–3.237, *P* = 0.941). This analysis is based on post-baseline measurements, which may introduce mediator or collider bias, and the results should be interpreted as hypothesis-generating only. Detailed results are presented in Supplementary Table S3.

## 4 Discussion

This single-center retrospective cohort study, after controlling for baseline confounding factors using propensity score matching, found that quadruple therapy was associated with a lower risk of the composite endpoint of heart failure hospitalization or cardiovascular death in patients with HFpEF. The observed association was primarily driven by a reduction in heart failure hospitalizations, while no statistically significant difference in cardiovascular or all-cause mortality was detected between groups. The primary endpoint result showed borderline statistical significance, and the findings should be interpreted with caution. Subgroup analyses further indicated that patients with concomitant coronary artery disease and baseline heart rate ≥70 beats per min showed a more pronounced association between quadruple therapy and reduced endpoint risk. An exploratory *post-hoc* analysis suggested a potential benefit in patients whose LVEF declined to 40%–49% during follow-up, but this finding is hypothesis-generating and requires further validation. The overall safety profiles were comparable between the two groups, with only a slightly higher incidence of mild sinus bradycardia in the quadruple therapy group, which was manageable with dose adjustment.

The efficacy of beta-blockers in HFpEF has long been controversial (20). The early SENIORS trial showed that nebivolol reduced all-cause mortality and cardiovascular hospitalization in elderly patients with heart failure, but subgroup analyses suggested that the benefit was primarily concentrated in patients with LVEF <50% (14). The CHARM-Preserved trial similarly found that the benefit of candesartan in patients with HFpEF was primarily derived from the subgroup with lower LVEF (15). A meta-analysis including 11 RCTs showed that beta-blockers reduced all-cause mortality but not heart failure hospitalization rates in patients with HFpEF (17). The main reason for the inconsistent results of these studies may lie in differences in foundational treatment regimens: most previous studies were conducted before SGLT-2i and ARNI became widely recommended for HFpEF, resulting in heterogeneous foundational treatments and significant confounding factors. A key strength of this study is that all included patients had received triple therapy (as defined per the study protocol) including SGLT-2i, and baseline characteristics between the two groups were rigorously balanced using the PSM method, minimizing confounding bias. Multiple real-world studies have reached similar conclusions to this study: a 2024 study based on the US Medicare database showed that the addition of a beta-blocker reduced the risk of the primary composite endpoint by 28% in patients with HFpEF receiving triple therapy (21); another meta-analysis also found that beta-blocker use was associated with lower rates of heart failure rehospitalization in patients with HFpEF (17). The results of this study further confirm that quadruple therapy still provides additional clinical benefits to patients with HFpEF in the current guideline-directed treatment era.

In the prespecified subgroup analysis based on baseline LVEF, no statistically significant heterogeneity in treatment effect was detected across strata, suggesting that baseline LVEF alone may not be a strong predictor of the magnitude of benefit from quadruple therapy. A trend toward greater association was observed in patients with baseline LVEF of 50%–59%, but this did not reach formal statistical significance. An exploratory *post-hoc* analysis using the lowest LVEF during follow-up suggested a more pronounced association in patients whose LVEF declined to 40%–49% during follow-up. However, this analysis is based on post-baseline LVEF values, which may be affected by disease progression and treatment itself, introducing potential mediator or collider bias. Therefore, this finding is hypothesis-generating and cannot be interpreted as a confirmatory conclusion. The potential mechanism underlying this observation may relate to more pronounced sympathetic nervous system activation in patients with declining LVEF, where quadruple therapy may exert a greater effect by inhibiting excessive sympathetic tone, slowing heart rate, and reducing myocardial oxygen consumption (22, 23). In contrast, patients with persistently preserved LVEF (≥60%) have pathophysiology dominated by myocardial fibrosis and primary diastolic dysfunction, in which sympathetic activation plays a relatively smaller role, potentially limiting the effect of beta-blockers (24). This finding requires further validation in prospective studies with serial LVEF assessments.

Furthermore, patients with concomitant coronary artery disease derived significant benefit from quadruple therapy, while no significant benefit was observed in patients without coronary artery disease. This result is consistent with the pharmacological characteristics of beta-blockers, which are cornerstone medications for secondary prevention of coronary artery disease. Beta-blockers improve myocardial ischemia and reduce the risk of myocardial infarction and sudden death by slowing heart rate, reducing myocardial contractility, and decreasing myocardial oxygen consumption (25). In patients with HFpEF and concomitant coronary artery disease, heart failure events are often closely related to myocardial ischemia, thus explaining the more pronounced benefit of quadruple therapy in this population. This finding generates a hypothesis that patients with concomitant coronary artery disease may derive more clinical benefit from quadruple therapy, which requires further validation in prospective, adequately powered studies.

Subgroup analysis stratified by baseline heart rate showed that patients with baseline heart rate ≥70 beats per min derived significant benefit from quadruple therapy, while no significant benefit was observed in patients with baseline heart rate <70 beats per min. This result echoes the findings of the SHIFT trial, which showed that patients with HFrEF and heart rate ≥70 beats per min derived the greatest benefit from ivabradine therapy (26). Although the SHIFT trial was conducted in patients with HFrEF, the benefit of heart rate control has also been demonstrated in multiple studies of HFpEF (27). Elevated heart rate is a marker of sympathetic activation, leading to shortened diastolic filling time, increased myocardial oxygen consumption, and exacerbation of myocardial ischemia and diastolic dysfunction. By slowing heart rate, these agents improve left ventricular diastolic filling and reduce myocardial oxygen consumption, thereby reducing the occurrence of heart failure events (28).

NT-proBNP is a sensitive biomarker reflecting left ventricular filling pressure and myocardial remodeling, and its changes are closely related to the prognosis of patients with heart failure (29). This study showed that NT-proBNP levels decreased more significantly in the quadruple therapy group, and the trend of NT-proBNP changes was consistent with the occurrence of clinical endpoint events: differences in NT-proBNP levels between the two groups began to emerge at 6 months of follow-up, reached statistical significance at 12 months, and further widened at 24 months. This result suggests that quadruple therapy may reduce the risk of heart failure events by improving left ventricular diastolic function and attenuating myocardial remodeling. Meanwhile, early changes in NT-proBNP can serve as an important indicator for evaluating the efficacy of this treatment strategy, providing a basis for clinical treatment adjustment.

In terms of safety, the results of this study showed that the overall safety of quadruple therapy was comparable to that of triple therapy, with no statistically significant differences between the two groups in the overall incidence of adverse events, serious adverse events, and treatment discontinuation rate due to adverse events. Only the incidence of sinus bradycardia was slightly higher in the quadruple therapy group than in the triple therapy group (6.7% vs. 2.2%, *P* = 0.041), but all bradycardia events were mild, asymptomatic, and resolved after beta-blocker dose adjustment of beta-blockers, with no episodes of severe bradycardia, cardiac arrest, or need for pacemaker implantation. There were no statistically significant differences between the two groups in the incidence of symptomatic hypotension, atrioventricular block, hyperkalemia, and acute kidney injury. These results indicate that quadruple therapy has a favorable safety and tolerability profile, with manageable clinical application risks.

This study has several limitations that should be acknowledged. First, this was a retrospective cohort study. Although PSM and IPTW were used to control for measured confounding factors including baseline heart rate, coronary artery disease status, blood pressure, and markers of disease severity, residual confounding and confounding by indication cannot be fully eliminated. In clinical practice, beta-blockers may be preferentially prescribed to patients with concomitant coronary artery disease, higher baseline heart rate, or more stable hemodynamic profiles, which may partially influence the observed association and introduce selection bias. Sensitivity analyses including IPTW and landmark analysis yielded consistent direction of effect, supporting the robustness of the primary findings, but unmeasured factors such as detailed medication adherence, lifestyle habits, socioeconomic status, and psychological status may still affect the precision of effect estimates. Negative control analysis was not performed in this study due to the lack of validated negative control outcomes in the retrospective dataset, which represents an additional limitation for confounding control. Additionally, as HFpEF diagnostic guidelines evolved over the study enrollment period (2018–2025), we applied the 2023 guideline criteria uniformly to all patients through retrospective adjudication to ensure cohort homogeneity. However, this approach may have introduced selection bias by excluding some patients diagnosed under earlier, less stringent criteria, and the generalizability of our findings to populations defined by other guideline versions warrants consideration. Second, this was a single-center study with a relatively limited sample size, and the sample size of some subgroups was small, particularly the subgroup with LVEF ≥60%, which included only 82 patients, potentially resulting in insufficient statistical power for subgroup analyses to detect potential efficacy differences. In addition, no adjustment for multiple testing was applied across subgroup analyses, and observed interaction effects may be influenced by chance. Subgroup results are not suitable for deriving clinical treatment recommendations and can only be used as clues for subsequent research. Third, this study did not use a randomized design, and selection bias cannot be completely excluded; clinicians may have been more likely to prescribe beta-blockers to patients with milder disease, higher heart rate, and concomitant coronary artery disease, although the PSM method balanced these factors to some extent, this bias cannot be completely eliminated. Fourth, dose titration of beta-blockers was determined by clinicians based on individual patient conditions, and there may be differences in treatment strategies and dose adjustment criteria among different physicians. The average dose titration rate to target in this study was 62.8%, which did not reach the levels achieved in RCTs, potentially underestimating the true efficacy of quadruple therapy. Fifth, patient-reported outcomes and functional parameters such as quality of life, 6-min walk distance, and exercise tolerance were not collected, precluding assessment of the impact of quadruple therapy on patient quality of life and functional status. Sixth, the study had limited statistical power to detect differences in rare but clinically important adverse events (e.g., severe atrioventricular block, severe bronchospasm) due to the relatively small sample size; thus, the long-term safety profile of quadruple therapy requires further verification in larger cohorts. Finally, the maximum follow-up duration was 72.3 months, with a median follow-up of 37.8 months, and longer-term follow-up data are lacking to evaluate the long-term efficacy and safety of quadruple therapy.

Despite these limitations, the results of this study provide supportive real-world evidence for the clinical use of quadruple therapy in selected patients with HFpEF. Given the observational design and borderline statistical significance of the primary endpoint, the findings are hypothesis-generating and cannot establish a causal relationship between quadruple therapy and improved outcomes. Future large-scale, multicenter, randomized controlled trials are needed to further validate the results of this study and explore more precise biomarkers to guide the individualized application of beta-blockers in patients with HFpEF.

## Data Availability

The original contributions presented in the study are included in the article/Supplementary Material, further inquiries can be directed to the corresponding author.

## Supplementary material

Supplementary Figure S1 Distribution of propensity scores before and after propensity score matching.

Supplementary Figure S2 Love plot displaying standardized mean differences (SMD) for all baseline covariates before and after propensity score matching.
