# Supplementary material for: Efficacy and safety of quadruple therapy versus triple therapy in patients with heart failure with preserved ejection fraction: a propensity score-matched real-world study
Source: Front Cardiovasc Med. 2026 Jul 20;13:1893975. doi: 10.3389/fcvm.2026.1893975 (PMC13429833; doi:10.3389/fcvm.2026.1893975)
Supplement: Supplementary Figure S1 — Distribution of propensity scores before and after propensity score matching. [file Datasheet1.zip › Supplementary materials/Supplementary Table 3.docx]

**Supplementary Table 3. Exploratory Post-hoc Subgroup Analysis by Follow-up Lowest LVEF.**

| **LVEF Stratum (Follow-up Lowest)** | **Events/Total (Quadruple)** | **Events/Total (Triple)** | **HR (95% CI)** | **Subgroup P-value** | **P for Interaction** |
| --- | --- | --- | --- | --- | --- |
| 40%–49% | 8/36 | 16/35 | 0.421 (0.183–0.976) | 0.044 | 0.028 |
| 50%–59% | 19/102 | 24/105 | 0.783 (0.426–1.453) | 0.437 |  |
| ≥60% | 5/42 | 5/40 | 0.954 (0.285–3.237) | 0.941 |  |

Note: This is an exploratory post-hoc analysis based on the lowest LVEF recorded during follow-up. Results are hypothesis-generating and subject to potential bias from post-baseline stratification.
