# Supplementary material for: Efficacy and safety of quadruple therapy versus triple therapy in patients with heart failure with preserved ejection fraction: a propensity score-matched real-world study
Source: Front Cardiovasc Med. 2026 Jul 20;13:1893975. doi: 10.3389/fcvm.2026.1893975 (PMC13429833; doi:10.3389/fcvm.2026.1893975)
Supplement: Supplementary Figure S1 — Distribution of propensity scores before and after propensity score matching. [file Datasheet1.zip › Supplementary materials/Supplementary Table 4.docx]

**Supplementary Table 4. Detailed Beta-Blocker Use Characteristics in the Quadruple Therapy Group.**

| **Parameter** | **Value (n=180)** |
| --- | --- |
| Beta-blocker type, n (%) |  |
| Metoprolol succinate extended-release | 112 (62.2) |
| Bisoprolol fumarate | 54 (30.0) |
| Carvedilol | 14 (7.8) |
| Baseline target-dose achievement, n (%) | 113 (62.8) |
| Mean daily dose (% of target dose), % | 71.4±22.6 |
| Dose down-titration during follow-up, n (%) | 21 (11.7) |
| Primary reason for down-titration, n (%) |  |
| Bradycardia | 9 (5.0) |
| Symptomatic hypotension | 5 (2.8) |
| Fatigue/asthenia | 4 (2.2) |
| Other | 3 (1.7) |
| Permanent beta-blocker discontinuation, n (%) | 9 (5.0) |
| Primary reason for discontinuation, n (%) |  |
| Symptomatic bradycardia | 3 (1.7) |
| Recurrent symptomatic hypotension | 2 (1.1) |
| Bronchospasm | 1 (0.6) |
| Other | 3 (1.7) |
| Treatment persistence at 12 months, % | 94.4 |

Note: All adverse events leading to dose adjustment were graded 1–2 according to NCI-CTCAE 5.0 criteria.
