# Supplementary material for: Efficacy and safety of quadruple therapy versus triple therapy in patients with heart failure with preserved ejection fraction: a propensity score-matched real-world study
Source: Front Cardiovasc Med. 2026 Jul 20;13:1893975. doi: 10.3389/fcvm.2026.1893975 (PMC13429833; doi:10.3389/fcvm.2026.1893975)
Supplement: Supplementary Figure S1 — Distribution of propensity scores before and after propensity score matching. [file Datasheet1.zip › Supplementary materials/Supplementary Table 1.docx]

**Supplementary Table 1. Standardized Mean Differences of Baseline Covariates Before and After Inverse Probability of Treatment Weighting.**

| **Characteristic** | **SMD Before IPTW** | **SMD After IPTW** |
| --- | --- | --- |
| Age (years) | 0.124 | 0.021 |
| Female | 0.053 | 0.011 |
| BMI (kg/m²) | 0.081 | 0.024 |
| Obesity (BMI≥30 kg/m²) | 0.088 | 0.023 |
| Smoking history |  |  |
| Current smoker | 0.051 | 0.014 |
| Former smoker | 0.058 | 0.012 |
| Never smoker | 0.018 | 0.007 |
| NYHA functional class |  |  |
| Class II | 0.108 | 0.035 |
| Class III | 0.099 | 0.034 |
| Class IV | 0.015 | 0.004 |
| HF duration (years) | 0.076 | 0.019 |
| Systolic blood pressure (mmHg) | 0.221 | 0.032 |
| Diastolic blood pressure (mmHg) | 0.279 | 0.028 |
| Heart rate (beats/min) | 0.039 | 0.010 |
| Hypertension | 0.056 | 0.016 |
| Type 2 diabetes mellitus | 0.099 | 0.027 |
| Coronary artery disease | 0.257 | 0.036 |
| Atrial fibrillation | 0.061 | 0.013 |
| Chronic kidney disease (eGFR<60 mL/min/1.73m²) | 0.085 | 0.025 |
| NT-proBNP (pg/mL) | 0.042 | 0.015 |
| eGFR (mL/min/1.73m²) | 0.079 | 0.022 |
| Hemoglobin (g/L) | 0.119 | 0.031 |
| Serum potassium (mmol/L) | 0.069 | 0.017 |
| Fasting blood glucose (mmol/L) | 0.052 | 0.011 |
| LDL-C (mmol/L) | 0.055 | 0.018 |
| LVEF (%) | 0.234 | 0.029 |
| Left atrial diameter (mm) | 0.098 | 0.024 |
| E/e' ratio | 0.080 | 0.026 |
| Left ventricular end-diastolic diameter (mm) | 0.054 | 0.015 |
| Peak tricuspid regurgitation velocity (m/s) | 0.089 | 0.023 |
| SGLT-2 inhibitors | 0.000 | 0.000 |
| ARNI/ACEI/ARB and MRA | 0.000 | 0.000 |
| Loop diuretics | 0.000 | 0.000 |
| Beta-blockers | 1.000 | 1.000 |
| Statins | 0.065 | 0.016 |
| Antiplatelet agents | 0.252 | 0.038 |
| Oral anticoagulants | 0.084 | 0.022 |

Note: SMD, standardized mean difference; IPTW, inverse probability of treatment weighting. SMD < 0.1 indicates negligible imbalance between comparison groups.
