# Supplementary figures and images for: Efficacy and safety of quadruple therapy versus triple therapy in patients with heart failure with preserved ejection fraction: a propensity score-matched real-world study
Source: Front Cardiovasc Med. 2026 Jul 20;13:1893975. doi: 10.3389/fcvm.2026.1893975 (PMC13429833; doi:10.3389/fcvm.2026.1893975)

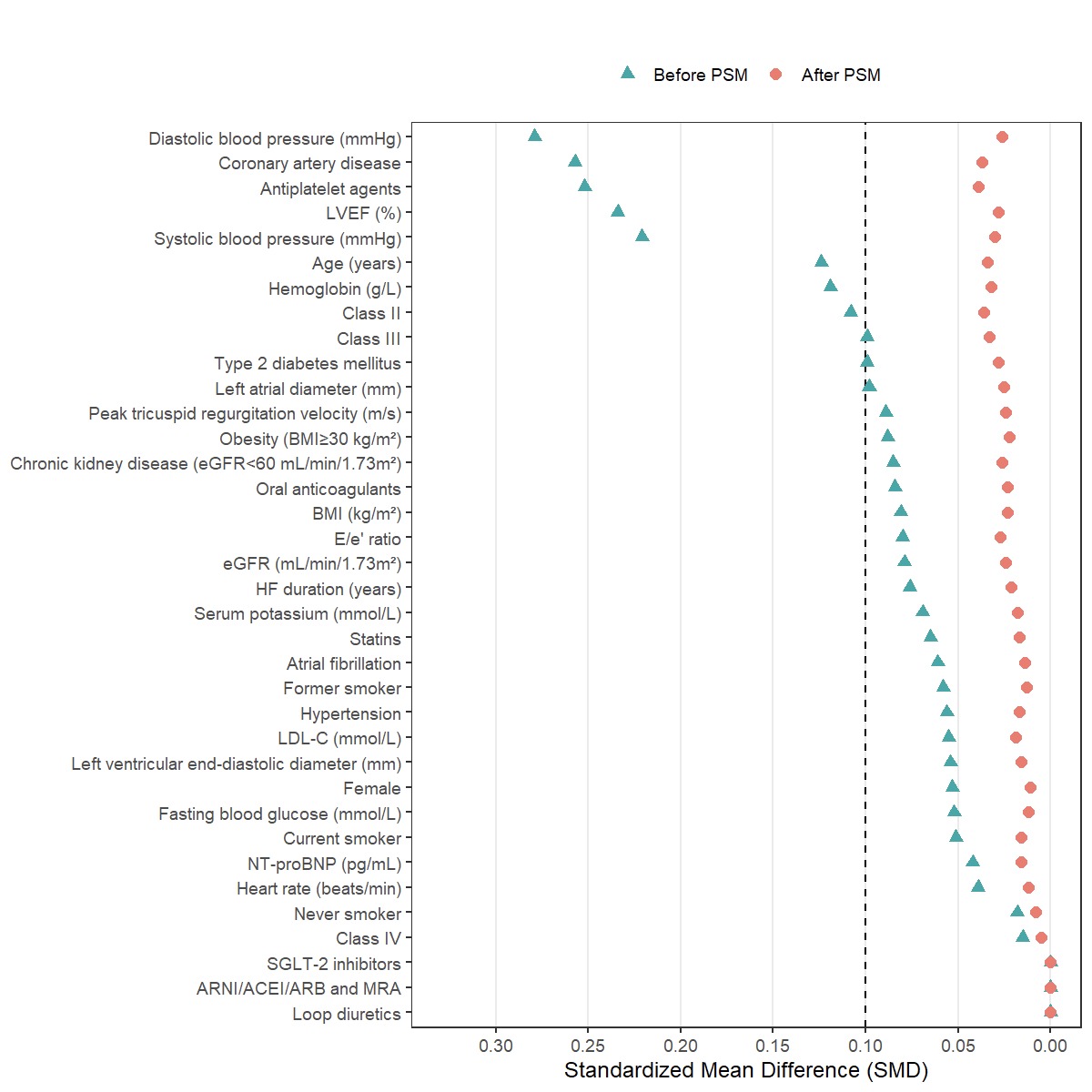

Supplement: Supplementary Figure S1 — Distribution of propensity scores before and after propensity score matching. [file Datasheet1.zip › Supplementary materials/Supplementary Figure 2.tiff]

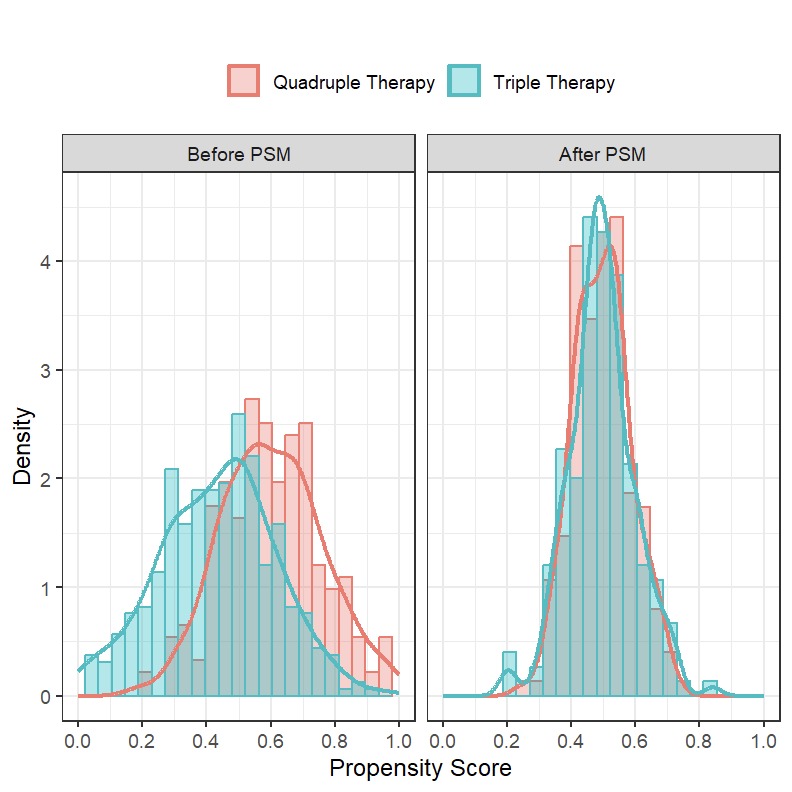

Supplement: Supplementary Figure S1 — Distribution of propensity scores before and after propensity score matching. [file Datasheet1.zip › Supplementary materials/Supplementary Figure 1.tiff]
